# Update on the distribution of *Mansonella perstans* in the southern part of Cameroon: influence of ecological factors and mass drug administration with ivermectin

**DOI:** 10.1186/s13071-016-1595-1

**Published:** 2016-05-31

**Authors:** Samuel Wanji, Dizzle Bita Tayong, Laura E. Layland, Fabrice R. Datchoua Poutcheu, Winston Patrick Chounna Ndongmo, Jonas Arnaud Kengne-Ouafo, Manuel Ritter, Nathalie Amvongo-Adjia, Fanny Fri Fombad, Charity Nya Njeshi, Armand Seraphin Nkwescheu, Peter A. Enyong, Achim Hoerauf

**Affiliations:** Parasite and Vector Research Unit (PAVRU), Department of Microbiology and Parasitology, University of Buea, Buea, Cameroon; Research Foundation for Tropical Diseases and the Environment (REFOTDE), Buea, Cameroon; Scientific Networks and Ethics Promotion, Division of Health Operations Research, Ministry of Public Health, Yaoundé, Republic of Cameroon; Institute of Medical Microbiology, Immunology and Parasitology (IMMIP), University Hospital of Bonn, Bonn, Germany; German Centre for Infection Research (DZIF), partner site, Bonn-Cologne, Bonn, Germany

**Keywords:** Mansonellosis, *Mansonella perstans*, Microfilariae, Distribution, Prevalence, Intensity, Bioecological zones, Ivermectin, MDA, Cameroon

## Abstract

**Background:**

Mansonellosis remains one of the most neglected of tropical diseases and its current distribution in the entire forest block of southern Cameroon is unknown. In order to address this issue, we have surveyed the distribution of *Mansonella perstans* in different bioecological zones and in addition, elucidated the influence of multiple rounds of ivermectin (IVM) based mass drug administration (MDA).

**Methods:**

A mixed design was used. Between 2000 and 2014, both cross-sectional and longitudinal surveys were carried out in 137 communities selected from 12 health districts belonging to five main bioecological zones of the southern part of Cameroon. The zones comprised of grassland savanna (GS), mosaic forest savanna (MFS), forested savanna (FS), deciduous equatorial rainforest (DERF) and the dense humid equatorial rainforest (DHERF). The survey was carried out in some areas with no treatment history as well as those currently under IVM MDA. Individuals within the participatory communities were screened for the presence of *M. perstans* microfilariae (mf) in peripheral blood by the calibrated thick film method to determine both prevalence and geometric mean intensities at the community level.

**Results:**

Apart from sporadic cases in savanna areas, distribution of *M. perstans* was strongly linked to the equatorial rainforest zones. Before CDTI, the highest mean prevalence (70.0 %) and intensity (17,382.2 mf/ml) were obtained in communities in Mamfes’ DHERF areas followed by communities in the DHERF zone of Lolodorf (53.8 % and 7,814.8 mf/ml, respectively). A longitudinal survey in Mamfe further showed that *M. perstans* infections had reduced by 34.5 % in DERF (*P* < 0.001) but not DHERF zones after ten years of IVM MDA. Further data from the cross-sectional study revealed that there was a decrease in prevalence in DHERF zones only after ten years of MDA. In DERF zones however, the infection was relatively lower after four years of MDA.

**Conclusions:**

The distribution of *M. perstans* in the southern part of Cameroon varies with bioecological zones and IVM MDA history. The zones with high prevalence and intensities lie in forested areas while those with low endemicity are in the savanna areas. MDA with ivermectin induced significant reduction in the endemicity of mansonellosis in the decidious equatorial rainforest. In contrast, the prevalence and intensity remained relatively high and stable in the dense humid equatorial rainforest zones even after a decade of mass drug administration with ivermectin. Since it is known that *M. perstans* down-regulates host's immune system, the findings from this work would be useful in designing studies to understand the impact of *M. perstans* on host immune response to vaccination and co-infection with other pathogens such as *Mycobacterium* spp. and *Plasmodium* spp. in areas of contrasting endemicities.

**Electronic supplementary material:**

The online version of this article (doi:10.1186/s13071-016-1595-1) contains supplementary material, which is available to authorized users.

## Background

*Mansonella perstans* (formerly *Dipetalonema perstans*) is a vector-borne human filarial nematode, transmitted by tiny blood-sucking midges of the genus *Culicoides* and is the causative agent of mansonellosis [1]. It is one of the most prevalent human parasites in sub-Saharan Africa (reported in 33 countries) and over 114 million people are infected throughout the continent [1]. On the west coast of Africa, it spreads from Senegal to Angola, through central Africa, north to southwest Sudan, east to Uganda, Kenya and Tanzania and south to Zimbabwe [2]. In most affected countries (Cameroon, Nigeria, Ghana, Sierra Leone, Ivory Coast, Zambia and Uganda), the disease occurs mainly among poor people living in rural villages [2, 3]. Although absent in most northern and southern regions of Africa and Asia, *M. perstans* infections are prevalent in Central and South America as well as the Caribbean [1].

Infections with *M. perstans* present mild pathology compared to infections with other filarial parasites such as *Wuchereria bancrofti*, *Brugia malayi*, *Onchocerca volvulus* and *Loa loa*. However, the pathogenicity of *M. perstans* infection has recently been reconsidered since Bregani et al. [4], documented that *M. perstans* has the ability to induce a variety of clinical features, including angioedema Calabar-like swellings, pruritus, fever, headache, high eosinophilia, abdominal pain, arthralgia, and neurologic manifestations. In addition, people harbouring the parasite have reported bouts of itchiness and dizziness [5] and there are reports that *M. perstans* is possibly involved in the development of ocular filariasis as well [6]. Infections with *M. perstans* have also been shown to induce an immunosuppressive environment in the host [1] through the involvement of a pleiotropic immunomodulatory cytokine IL-10 [7, 8]. IL-10 is well known for its ability to down regulate T helper 1-type responses such as IFN-gamma secretion and activation of monocytes/macrophages [9] and as such, the host may become vulnerable to other pathogens gaining access and invading a dampened immune system*.*

No specific treatment exists for mansonellosis and ivermectin (IVM), widely used for mass drug administration (MDA) for other filariae, has been reported to have little effect on the infection through community directed treatment with ivermectin (CDTI) [10]. Mebendazole has also been shown to have moderate effect on *M. perstans* [11]. Although rainforest areas and warm climates seem to favour *M. perstans*, a detailed knowledge about the environmental factors that support the vector and may account for the differential distribution of the disease is limited [12, 13], especially in Cameroon. In 1995, Mommers et al. [13] provided an account of the distribution of *M. perstans* in the southern part of Cameroon; however, prevalence in entire forest block remains unclear since repeated surveys were only performed in a single community. Due to the relevance of this parasite in down-regulating host immunity, we have surveyed the distribution and prevalence of *M. perstans* infection in major bio ecological zones (grassland savannah, mosaic forest savannah, forested savannah, deciduous equatorial rainforest and dense humid equatorial rainforest) in southern Cameroon. Moreover, we compared prevalence of infection in areas before and > 10 years after IVM MDA introduction. These datasets provide useful tools for designing studies aimed at understanding the impact of *M. perstans* on host immunity against other pathogens such as *Mycobacterium* species.

## Methods

### Study design

**Fig. 1:**
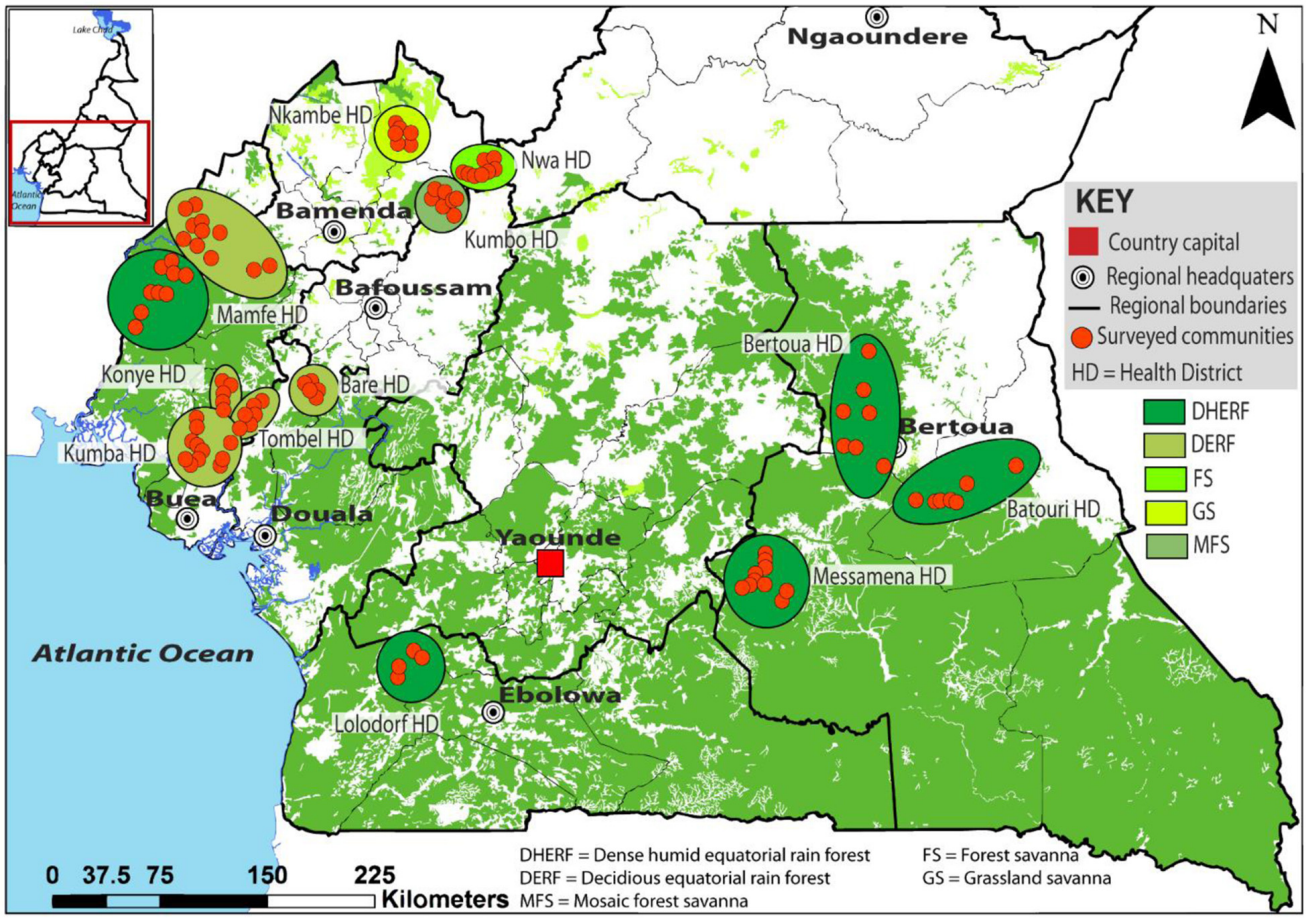
Bioecological zones in *M. perstans* endemic areas in cameroon. Map shows sampling areas in relationship to bioecological zones. FS, forest savannah; MFS, mosaic forest savannah; GS, grassland savannah; DERF, deciduous equatorial rainforest; and DHERF, dense habitat equatorial rainforest

**Fig. 2:**
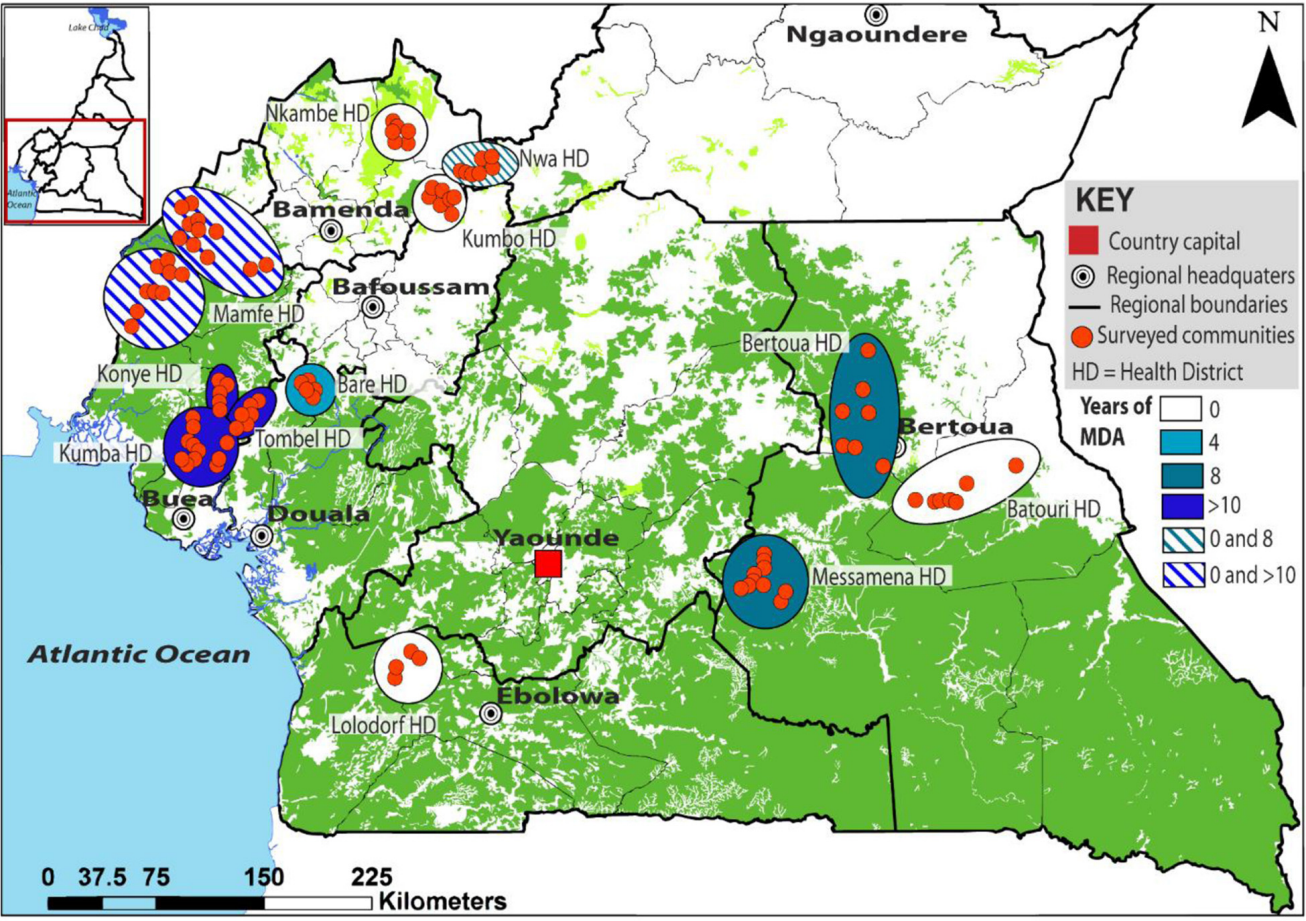
Rounds of mass drug administration programmes in years in *M. perstans* endemic areas in Cameroon. MDA rounds at the time of sampling in 2013: white = no treatment; pale blue = 4 years; mid-blue = 8 years MDA and dark blue = >10 years. Striped areas refer to sites of longitudinal survey where in, samples were obtained before MDA started and after either 8 (Nwa) or >10 (Mamfe) years

This study used a mixed study design (cross-sectional and longitudinal surveys) and was carried out in 137 communities of five main bioecological zones: grassland savannah (GS), mosaic forest savannah (MFS), forested savannah (FS), deciduous equatorial rainforest (DERF) and dense humid equatorial rainforest (DHERF), belonging to 12 health districts of Cameroon from the year 2000 to 2014 (Fig. 1). Based on the stratification of health districts, communities were randomly selected as well as households within the communities. To establish the possible impact of IVM MDA on *M. perstans* distribution, the health districts in different bioecological zones were classified into three main groups. The first group was health districts surveyed before the introduction of IVM MDA or with no treatment history at the time of sampling. The second group was health districts surveyed whilst IVM-MDA has been ongoing and the third group was specific communities of health districts (Mamfe and Nwa) surveyed before and whilst under IVM-MDA. Figure 2 shows in detail, the current years of IVM-MDA in each surveyed health district. In each participatory community, eligible individuals (both males and females) were those that had lived in the communities for at least five years and were at least five years old. Recruited volunteers that met the eligibility criteria gave their full written consent and for children, consent was provided by the child's parent or legal guardian. Details on gender and median age of individuals in the various health districts and bioecological zones are shown in Additional file 1: Figure S1. Each volunteer was screened for the presence of *M. perstans* microfilariae in peripheral blood. Both prevalence and geometric mean intensities were generated with respect to communities in health districts and statistically compared for the different bioecological zones.

### Study site

The study was carried out in communities selected from both rainforest and savanna areas in Cameroon. The climate varied from "Guinea type" in the South and East bioecological zones through equatorial hot and wet in the Littoral, to temperate in the South West and humid savanna/tropical in the North West zones. Warm climates (guinea and temperate type) seem to favour the transmission of *M. perstans.* The climate of savanna areas are typically humid savanna to tropical forest type and in this study we surveyed three distinct savanna regions. GS: this includes communities of the Nkambe health district of north-west Cameroon. This area is mainly grassland, characterised by sparse trees that are widely spaced to provide an open canopy. Areas of MFS include communities of the Kumbo health district still in the western highlands of Cameroon. The vegetation is mainly grassland crossed with trees growing alongside streams and on hillsides but the tree density is less compared to the forested savanna. FS contained communities of the Nwa health district also in the western highlands of Cameroon. The vegetation here is typically grass with a very high density of trees and has a humid climate. This FS is thus a transition between savanna and forest zones. DERF areas comprised communities in Bare, Tombel, Konye, Kumba and Mamfe health districts in the littoral and south-west regions of Cameroon. This type of forest has a warm climate and is characterised with high rainfalls while the dry season is cooler. Also many trees shed their leaves creating a reduction in the canopy thus permitting sunlight to reach the forest floor and hence growth of vigorous ground vegetation [14]. Finally, the DHERF comprised some communities of Mamfe, Bertoua, Messamena, Batouri and Lolodorf health districts in the south-west, south and east regions of Cameroon. The climatic conditions remain equal here all year round and are marked with high daily and nightly temperatures as well as heavy daily rainfall. The warmth and high humidity also accounts for the high biological diversity observed in these areas. Unlike the DERF, no leaves are shed here in the dry season, and as such, the DHERF have a well-developed canopy "tier" form of vegetation [14] and can be referred to as primary forests. The majority of the inhabitants of these zones are subsistence farmers and the main cash crops include cocoa, rubber, cassava, maize, yams, plantains and bananas. Crops like bananas and plantains as well as their products are cultivated near homes and serve therefore as efficient breeding sites for *Culicoides* spp. As such, the population is exposed to the bites of the vectors through their activities.

### Parasitological screening

Diagnosis of mansonellosis is through the detection and identification of *M. perstans* mf in peripheral blood [15]. Samples were collected for diagnosis during the day. Thick blood films were prepared from standardized 50 μl finger pricked blood using 75 μl non-heparinised capillary tubes. Smears were prepared by spreading the blood on clean slides covering an area of 1.5 × 2.5 cm and allowed to air-dry. In the laboratory, the blood smears were dehaemoglobinised using tap water for 10 to 15 min and were fixed with methanol for 1 min. Smears were later stained in 10 % Giemsa for 45 min, rinsed in distilled water and allowed to air-dry [3, 16]. Slides were read under a light microscope at a magnification of 10× by trained technicians. Parasites were identified using the mf identification keys of Orihel et al. [17]. In study areas co-endemic for *Loa loa*, another filarial species with blood dwelling mf, identification was done by two well-trained technicians in a blind manner. Mf counts were expressed as microfilariae per millilitre of blood (mf/ml).

### Data analysis

Statistical analyses were performed using the software SPSS (IBM SPSS Statistics 20; Armonk, NY) and GraphPad PRISM version 5.02 for Windows (GraphPad Software, Inc., La Jolla, USA, www.graphpad.com). *P*-values of less than 0.05 were considered statistically significant. For data that were not normally distributed, the following tests were performed: Kruskal-Wallis test was performed to compare three groups, followed by a Mann-Whitney U-tests for further pairwise comparisons. For comparisons of continuous parameters, the Spearman rank correlation was used. Mann-Whitney and Kruskal-Wallis tests were used to compare mean prevalence and geometric mean intensity of *M. perstans* in communities and health districts of the different surveyed bioecological zones. Mf prevalence and geometric mean intensities were also compared with respect to MDA history.

## Results

### Absence of IVM-MDA programmes reveals a dominant *M. perstans* prevalence in ERF areas

**Fig. 3:**
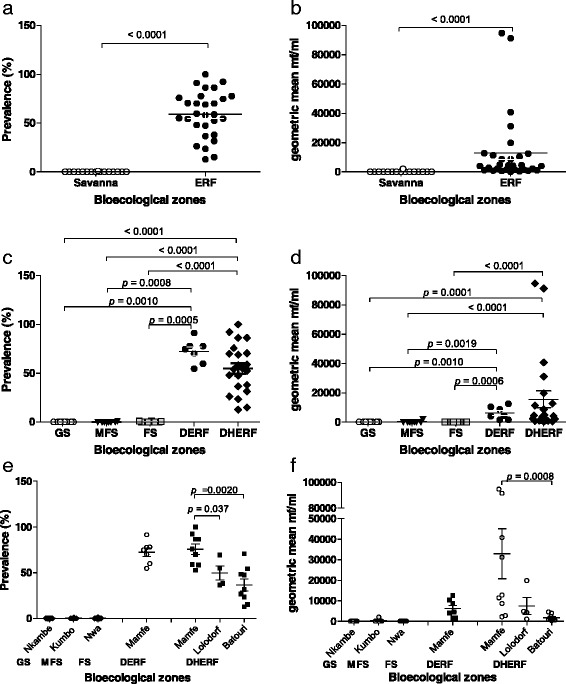
*M. perstans* infections are prevalent in ERF but not savanna areas. To determine the baseline prevalence (**a**) and intensity (**b**) of *M. perstans* infection prior to MDA in South West and eastern districts of Cameroon, individuals were screened in savannah (*n* = 3,073) and ERF (*n* = 2,648) zones that were yet to begin MDA programmes. Prevalence (**c**) and associated mf counts (**d**) were then assessed in GS, MFS and FS savannah districts and DERF and DHERF forest districts. Data were then further examined on an individual district level: Nkambe, GS (8 communities/*n* = 1,167); Kumbo, MFS (8 communities/*n* = 878); Mamfe, DERF (7 communities/*n* = 818) and DHERF (9 communities/*n* = 640); Lolodorf, DHERF (4 communities/*n* = 290), Batouri, DHERF (9 communities/*n* = 900); Nwa, FS (10 communities/*n* = 1,028). Statistical significance of the differences between groups indicated by the brackets were obtained after ANOVA/Kruskal-Wallis or Student's *t*-test or Mann-Whitney analysis depending on the normal distribution of the data

In this study, we analysed blood samples for *M. perstans* infection from individuals living in savanna (*n* = 3,073 individuals in 26 communities) and ERF (*n* = 2,648 individuals in 29 communities) areas that had not yet started ivermectin MDA programmes (Figs. 1 and 2). Figure 3a, b shows that the prevalence of *M. perstans* and corresponding mf intensity were significantly higher in ERF geographical areas since very few positive persons were detected in savanna districts (*n* = 3/3,073). We further subdivided the study area into savanna zones: grassland (GS), forest (FS) and mosaic forest (MFS), and equatorial rainforest (ERF) zones: deciduous equatorial rainforest (DERF) and dense humid equatorial rainforest (DHERF). This revealed that all savanna bioecological zones (MFS, FS and GS) were extremely low in infection whereas both DERF and DHERF zones had high prevalence, up to 100 % in some communities (Fig. 3c) and high mf intensity (Fig. 3d). Further analysis on individual health districts revealed that amongst the health districts in the DHERF region, Mamfe had a significantly higher prevalence of *M. perstans* infection than Lolodorf or Batouri (Fig. 3e). Significant differences in mf intensities were also revealed upon comparison of Mamfe and Batouri DHERF districts (Fig. 3f). No significant differences were found between Mamfe communities residing in DERF and DHERF areas. Thus, in the absence of any MDA programmes *M. perstans* infection is restricted to ERF geographical zones. Table 1 provides details about the prevalence and mf intensity in each surveyed community.

**Table 1:** *M. perstans* prevalence and intensity in five bioecological zones surveyed without any treatment history

Ecological zone	Health district/year of survey	Community	Number surveyed	Number positive	Prevalence (%)	Geometric mean (mf/ml)
		Bansobi	195	0	0	0
		Dzeng	186	0	0	0
		Kifem I	151	0	0	0
GS	Nkambe/2000	Lam	140	0	0	0
		Njanawa	105	0	0	0
		Rifem	212	0	0	0
		Tanyar	52	0	0	0
		Yang	126	0	0	0
		Total	1,167	0	0	0
		Bem	119	0	0	0
		Chunghe	78	0	0	0
		Kamine	125	0	0	0
		Kibbo	94	1	1.06	2,072.8
MFS	Kumbo/2000	Lip	133	0	0	0
		Mbissa	115	0	0	0
		Mfume	68	0	0	0
		Nkanchi	146	0	0	0
		Total	878	1	0.11	2,072.8
		Jator	111	0	0.0	0
		Mbiripkwa	70	0	0.0	0
		Ngomkow	66	0	0.0	0
		Ngu	112	1	0.9	13.90
		Nguri	90	0	0.0	0
FS	Nwa/2000	Nking	96	0	0.0	0
		Ntem	95	0	0.0	0
		Nwanti	107	0	0.0	0
		Nwat	52	0	0.0	0
		Sabongari	229	1	0.4	13.90
		Total	1,028	2	0.2	13.90
		Assam	57	52	91.2	10,402.8
		Bache	128	70	54.7	1,539.0
		Kesham	174	104	59.8	1,543.6
DERF	Mamfe/2000	Obonyi 1	121	94	77.7	1,2577.0
		Obonyi-3	159	119	74.8	4,036.1
		Okpambe	45	35	77.8	8,743.4
		Takamanda	134	94	70.1	4,427.3
		Total	818	568	69.4	4,344.7
		Ajaman	88	76	86.4	91,380.1
		Akwa	66	57	86.4	40,762.6
		Araru	30	21	70.0	2,199.2
		Babi	26	26	100.0	31,180.4
DHERF	Mamfe/2000	Babong	181	95	52.5	2,735.4
		Ekoneman	24	14	58.3	12,749.6
		Mbofong	39	36	92.3	94,757.9
		Nkoghau	107	63	58.9	11,391.1
		Ogurang	79	60	75.9	8,699.7
		Total	640	448	70.0	17,382.9
		Dem2	115	27	23.5	348.2
		Djal	114	30	26.3	1,380.9
		Kamba Mieri	126	19	15.1	811.6
		Konga	149	105	70.5	3,930.3
DHERF	Batouri/2013	Gabaleta	47	6	12.8	899.4
		Ngoulmekong	96	52	54.2	2,334.0
		Nguikouassima	63	20	31.7	589.1
		Baktala	110	53	48.2	1,226.7
		Bouam	80	38	47.5	4,573.6
		Total	900	350	38.9	1,947.7
		Bibondi	100	55	55.0	4,296.8
DHERF	Lolodorf/2010	Bikalla	94	65	69.1	19,797.5
		Bikoka	30	11	36.7	1,049.4
		Ngovayang	66	25	37.9	4,905.5
		Total	290	156	53.8	7,814.8

*Abbreviations*: *GS* grassland savanna, *MFS* mosaic forest savanna, *FS* forested savanna, *DERF* deciduous equatorial rainforest, *DHERF* dense humid equatorial rainforest, *mf/ml M. perstans* microfilariae per millilitre of blood

### *Mansonella perstans* prevalence and intensity in areas surveyed under MDA programmes reveal varying trends

Tables 2 and 3 show the current prevalence and intensity in ERF and FS districts that have had multiple rounds of IVM-MDA. With health districts surveyed under IVM-MDA for 4 and 8 years (Table 2), the prevalence ranged from 0.5 % in the FS of Nwa (8 years of IVM-MDA) through 23.2 % in the DERF of Bare (4 years of IVM-MDA) to 51.0 % in the DHERF of Messamena (8 years of IVM MDA). The same trend was observed in the mf geometric mean intensity. Therefore, both prevalence and intensity of *M. perstans* infection were highest in the DHERF compared to DERF and FS in communities surveyed 4 and 8 years under IVM-MDA. Whereas, with health districts surveyed with over a decade of treatment (Table 3), the highest prevalence was observed in the DERF of Mamfe (49.73 %) and the least in the DERF of Kumba (7.9 %). The mf intensity was rather high in the DHERF of Mamfe compared to the other health districts (Additional file 2).

**Table 2:** Prevalence and mean mf intensity of *M. perstans* in zones under CDTI for 4–8 years (Bare, Bertoua, Messamena and Nwa)

Ecological zone	Health district/year of survey	CDTI Status	Community	Number examined	Number positive	Prevalence (%)	Geometric mean (mf/ml)
			Mandjibo	67	11	16.4	209.6
			Mbarembeng I	95	4	4.2	62.6
			Mbarembeng II	72	3	4.2	29.2
			Mbie	27	12	44.4	502.6
		4 years	Mounko	67	3	4.5	85.6
DERF	Bare/2010		Mpaka	73	1	1.4	13.9
			Ndom	103	36	35.0	1,410.3
			Ndouembot	22	17	77.3	15,322.1
			Ndouenke	29	21	72.4	7,965.2
			Nkoniambot	174	51	29.3	314.9
			Nkoniankoniama	19	14	73.7	3,546.0
			Nkounianke	124	29	23.4	912.5
			Total	872	202	23.2	1,116.7
			Bombi	112	82	73.2	4,162.3
			Deng-Deng	105	40	38.1	3,354.1
DHERF	Bertoua/2013	8 years	Kanda	126	33	26.2	5,341.8
			Mbethen 2	112	86	76.8	3,193.2
			Ndemba 1	106	40	37.7	767.8
			Total	561	281	50.1	3,062.6
			Aviation	114	22	19.3	222.5
			Bissoua 2	155	107	69.0	3,758.8
			Doume Village	42	10	23.8	209.4
			Koum	45	26	57.8	898.4
			Labba	34	27	79.4	9,189.4
DHERF	Messamena/2013	8 years	Mayos	74	39	52.7	1,757.1
			Meba	47	35	74.5	2,532.2
			Messamena Village	78	46	59.0	5,275.1
			Nkomzuh	58	31	53.4	1,180.3
			Ntollock	52	14	26.9	75.9
			Soleye	163	83	50.9	1,976.9
			Total	862	440	51.0	2,115.8
			Jator	115	2	1.7	150.0
			Mbiripkwa	137	0	0	0
			Ngomkow	156	1	0.6	316.0
			Ngu	143	0	0	0
FS	Nwa/2013	8 years	Nguri	143	0	0	0
			Nking	108	0	0	0
			Ntem	153	0	0	0
			Nwanti	140	1	0.7	111.4
			Nwat	124	1	0.8	13.9
			Sabongari	110	1	0.9	1,509.8
			TOTAL	1329	6	0.5	175.8

*Abbreviations*: *GS* grassland savanna, *MFS* mosaic forest savanna, *FS* forested savanna, *DERF* deciduous equatorial rainforest, *DHERF* dense humid equatorial rainforest, *mf/ml M. perstans* microfilariae per millilitre of blood

**Table 3:** Prevalence and mean mf intensity of *M. perstans* in the DERF and DHERF surveyed after over a decade under CDTI (Mamfe, Konye, Kumba and Tombel health districts)

Ecological zone	Health district/year of survey	CDTI status	Community	Number examined	Number positive	Prevalence (%)	Geometric mean (mf/ml)
			Assam	59	40	67.80	4,763.3
DERF	Mamfe (2010 & 2013)	>10 years	Bache	104	47	45.20	1,146.1
			Kesham	126	55	43.7	972.5
			Okpambe	87	45	51.70	3,173.6
			Total	376	187	49.73	1,934.8
			Afap	26	5	19.20	21.8
			Ayukaba	116	12	10.34	1,128.1
			Babong	93	68	73.11	4,971.1
			Bokwa	60	20	33.33	274.3
			Ebam	95	2	2.11	801.3
			Eshobi	90	36	40.0	1,154.9
DHERF	Mamfe (2010 & 2013)	>10 years	Eyanchang	113	1	0.97	4,161.2
			Kajifu	100	22	22.0	3,101.8
			Mbakem	40	4	10.0	508.7
			Mbatop	115	9	7.83	299.7
			Mbeme	85	17	20.0	1,149.3
			Momboh	88	9	10.22	112.2
			Nkoghau	60	38	63.33	1,795.4
			Nyang	32	22	68.75	7,976.6
			Ogurang	86	66	76.74	12,170.2
			Taboh	77	11	14.30	73.1
			Total	1276	342	26.80	2,445.1
			Baduma	84	7	8.3	369.3
			Bakolle	78	5	6.4	155.7
			Bolo	107	11	10.3	165.6
			Dikomi Bafaw	101	12	11.9	72.6
			Eboko Bajor	89	10	11.2	216.7
DERF	Konye (2010 & 2013)	>10 years	Kokaka	74	14	35.1	122.1
			Kombone	97	11	11.3	204.3
			Kurume	45	5	11.1	86.0
			Matondo	107	5	4.7	206.9
			Weme	103	17	16.5	385.6
			Total	885	97	11.0	183.2
			Bai Bikom	91	0	0	0
			Bai Manya	89	2	2.2	150.0
			Bai Panya	43	0	0	0
			Bakumba	76	11	14.5	971.0
			Bambele	66	11	16.7	377.0
			Big Massaka	157	7	4.5	2,234.2
			Bikoki	46	30	65.2	1,077.6
			Boa Bakundu	60	6	10	451.7
DERF	Kumba (2010 & 2013)	>10 years	Bobanda	32	4	12.5	1,651.5
			Ediki	101	8	7.9	85.9
			Kotto Barombi	66	0	0	0
			Kumu-Kumu	34	11	32.4	418.8
			Marumba I	50	0	0	0
			Marumba II	68	0	0	0
			Mbalangi	100	2	2.0	359.1
			New Town	96	3	3.1	316.0
			Pete Bakundu	34	0	0	0
			Small Massaka	33	3	9.1	1,586.6
			Total	1241	98	7.9	667.7
			Bulutu	86	17	19.8	148.2
			Ehom	74	6	8.1	70.8
			Kack	142	28	19.7	337.5
			Mbabe	68	20	29.4	2,610.1
			Mbule	73	37	50.7	2,110.6
			Mile 18	54	6	11.1	279.5
DERF	Tombel (2010 & 2014)	>10 years	Mpako 1	63	21	33.3	1,052.8
			Ndom	67	18	26.9	609.2
			Ngab	53	14	26.4	392.9
			Ngusi	102	11	10.8	2,452.9
			Nlog	107	38	35.5	4,003.6
			Nsuke	113	18	15.9	1,646.2
			Peng	81	22	27.2	616.8
			Ndabekom	87	25	28.7	1,321.1
			Total	1,170	281	24.0	1,081.0

*Abbreviations*: *GS* grassland savanna, *MFS* mosaic forest savanna, *FS* forested savanna, *DERF* deciduous equatorial rainforest, *DHERF* dense humid equatorial rainforest, *mf/ml M. perstans* microfilariae per millilitre of blood

### Longitudinal surveys in Mamfe reveals reduced *M. perstans* infection in DERF but not DHERF zones after > 10 years IVM-MDA

**Fig. 4:**
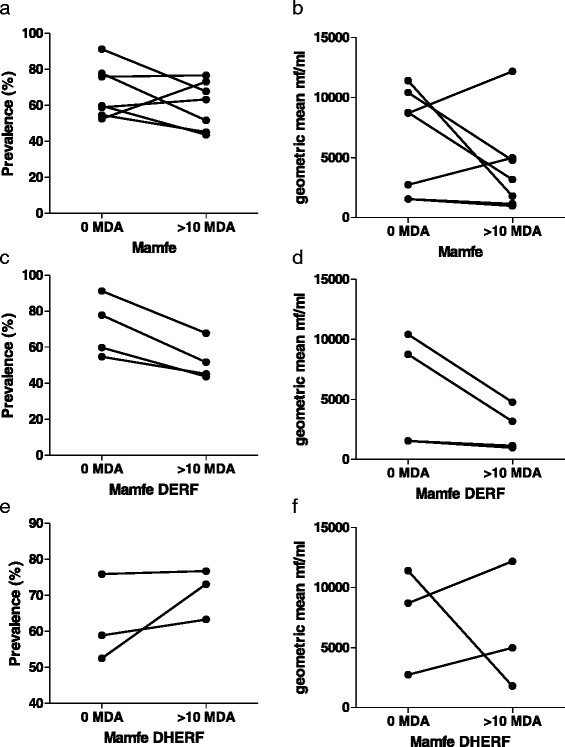
Longitudinal surveys in Mamfe reveal dampened *M. perstans* infection in DERF but not DHERF zones after >10 years MDA. **a** Prevalence and **b** mf intensity in Mamfe district (7 communities) prior to IVM therapy (0 MDA; *n* = 771) and after at least 10 years of MDA treatment (>10 MDA; *n* = 615). Analysis of individuals in DERF located communities of Mamfe (Assam, Bache, Kesham and Okpambe) revealed reduced prevalence and mf intensity **c** and **d** respectively): 0 MDA; *n* = 404 and > 10 MDA; *n* = 376. Infection in individuals of the DHERF communities of Mamfe (Babong, Ogurang and Nkoghau) remained unchanged or trended upwards (e and f): 0 MDA; *n* = 367 and > 10 MDA; *n* = 239. Symbols at each time point represent mean prevalence (%) or mf intensity (mf/ml) from one community

As shown in Fig. 3, in the absence of IVM-MDA *M. perstans* infections were highly prevalent in ERF zones. The district of Mamfe in the South West of Cameroon is an ERF geographical zone and we performed a longitudinal study of *M. perstans* infection between 2000 and 2013. Figure 4a, b provides comparative data for the prevalence and mf counts in Mamfe before the start of IVM-MDA (0 MDA; *n* = 771 individuals) and after more than 10 years of therapy (>10 MDA; *n* = 615 individuals). Data from these seven communities showed both trends of decrease and increase, therefore we divided the communities into those residing in DERF and DHERF zones (Fig. 1). Interestingly, communities residing in DERF zones (Assam, Bache, Kesham and Okpambe) presented a decreased prevalence (Fig. 3c) and mf intensities (Fig. 4d) whereas those in DHERF zones (Babong, Oguarag and Nkoghau) did not (Fig. 4e, f). Tables 1 and 3 show the data for each community before and after IVM-MDA treatment. Table 4 shows the percentage reduction in both prevalence and mf intensity of *M. perstans* in the communities of Mamfe in DERF and DHERF zones. In Fig. 3e, f we have also shown that the FS geographical zone Nwa had very low *M. perstans* infection prior to MDA (0.2 %) and in 2013, after > 8 years of MDA only 6/1,329 individuals were found microfilaraemic (Additional file 2: Figure S2; Additional file 3 Table S1). These data reveal two important situations: (i) FS zones contain little *M. perstans* and this has not changed before or after MDA treatment; and (ii) IVM treatment appears to have an effect on *M. perstans* infections in DERF but not DHERF zones (Additional file 2: Figure S2; Additional file 3 Table S1).

**Table 4:** Percentage reduction in *M. perstans* prevalence and intensity in selected communities of Mamfe and Nwa surveyed before and under CDTI MDA

Regions	Bioecological zones	Health district	Prevalence (%)					Geometric mean			
			Before IVM MDA	Under IVM MDA	% reduction	*χ* ^2^	*P*-value	Before IVM MDA	Under IVM MDA	% reduction	*P*-value
South west	DERF	Mamfe	64.60	49.73	23.02	0.7529	0.335	2,957.8	1,934.8	34.58	< 0.0001
	DHERF		59.40	71.97	-21.16	10.6411	0.001	5,800.9	5,743.6	0.99	< 0.0001
North west	FS	Nwa	0.20	0.50	-150.00	0.0331	0.281	13.90	175.8	-1164.74	< 0.0001

*Abbreviations*: *DERF* deciduous equatorial rainforest, *DHERF* dense humid equatorial rainforest, *FS* forested savanna, *IVM MDA* ivermectin mass drug administration (*χ* ^*2*^ *test* was used to compare prevalence rates before and under treatment; *t*-test was used to compare mean intensity of infection before and under treatment)

### Comparisons of DHERF communities following multiple MDA rounds reveals reduced prevalence in the south-western but not eastern Cameroon

**Fig. 5:**
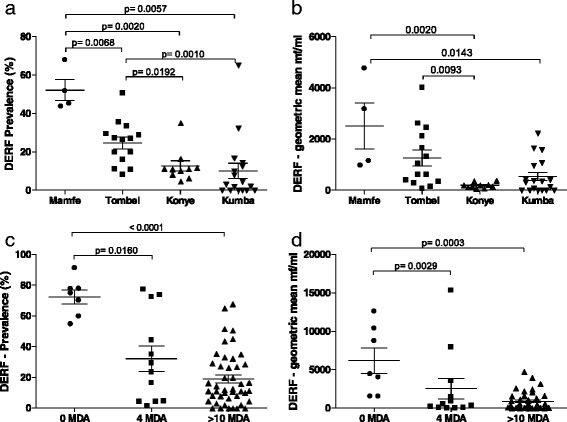
Reduced prevalence of *M. perstans* infection following multiple rounds of IVM-MDA in Mamfe DHERF district but not in DHERF areas in the East. **a**, **b** Prevalence of *M. perstans* infection in community members residing in DHERF zones prior to MDA and after at least 8 or 10 years IVM therapy. In 2013, neither Lolodorf (*n* = 290) nor Batouri (*n* = 900) districts in eastern Cameroon had entered in MDA programmes. Messamena (*n* = 862) and Bertoua (*n* = 561) districts, also in the East, had received at least 8 rounds of MDA and the South West district of Mamfe had had > 10 rounds MDA (*n* = 1,276). Mamfe 0 refers to prevalence and intensity prior to MDA therapy (*n* = 640). **c**, **d** show mf counts in the different communities based on rounds of MDA (0, 8 > 10) or per district respectively. Statistical significance of the differences between groups indicated by the brackets were obtained after ANOVA/Kruskal-Wallis or Student's *t*-test/Mann-Whitney analysis depending on the normal distribution of the data

Since *M. perstans* prevalence and intensities were different in DERF *vs* DHERF communities in Mamfe (Fig. 4), we further compared *M. perstans* infections in other DHERF areas that had had zero (Lolodorf and Batouri), 8 (Messemena and Bertoua) or > 10 rounds of MDA (Mamfe). Interestingly, prevalence and intensities were not significantly altered between 0 or 8 rounds of MDA but after more than 10 years, *M. perstans* infection rates were significantly reduced (Fig. 5a, b). To rule out that the observed reduction in Mamfe after 10 years was not due to simply lower prevalence to begin with, we also compared Mamfe levels prior to MDA and here, prevalence and mf levels were significantly higher than in 2013 (Fig. 5b, c). Moreover, prevalence rates in "Mamfe 0" were also significantly higher than in Lolodorf, Batouri and Messamena as well (Fig. 5b, *P* < 0.001). With regards to mf levels however, only individuals in Lolodorf showed significantly more mf/ml than those in Mamfe (Fig. 5d, *P* < 0.001) (Additional file 2).

### Communities within DERF zones show lower prevalence and intensity after multiple IVM-MDA rounds

**Fig. 6:**
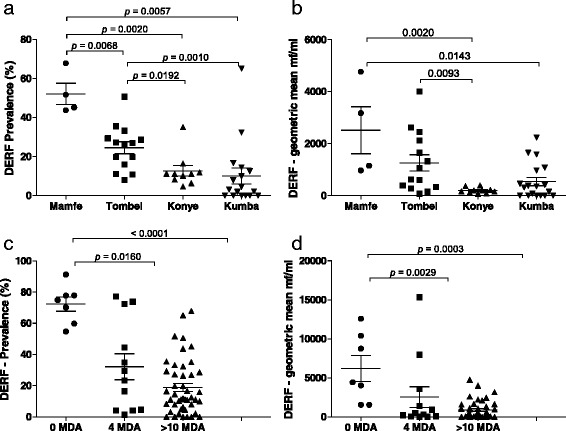
Kumba district shows the lowest prevalence of *M. perstans* infection in DERF bioecological zones. **a**, **b** show prevalence and mf intensity in *M. perstans* infected individuals living in DERF bioecological zones after at least 10 years of MDA therapy. Mamfe (*n* = 376); Tombel (*n* = 1,170); Konye (*n* = 885); and Kumba (*n* = 1,241). **c**, **d** show the prevalence and mf numbers in individuals living in DERF zones that had had 0 MDA (Mamfe; *n* = 404), > 4 years of MDA (Bare; *n* = 872) and > 10 years of MDA (Tombel, Konye, Kumba and Mamfe; *n* = 3,672). Statistical significance of the differences between groups indicated by the brackets were obtained after ANOVA/Kruskal-Wallis or Student's *t*-test/Mann-Whitney analysis depending on the normal distribution of the data

As depicted in Fig. 1, Mamfe can be divided into DERF and DHERF zones. Since *M. perstans* prevalence and intensity had decreased in the DHERF Mamfe health district after > 10 years of IVM-MDA (Fig. 5a, c), we further compared the effects of > 10 years IVM-MDA on *M. perstans* in communities in DERF zones. Here, communities in Mamfe (*n* = 4), Tombel (*n* = 14), Kumba (*n* = 18) and Konye (*n* = 10) were compared. Interestingly, the highest prevalence of *M. perstans* was found in individuals living in Mamfe (Fig. 6a) and mf intensities were also significantly higher when compared to Konye and Kumba (Fig. 6b, *P* <0.001). Prevalence rates in communities within Tombel were also significantly higher than those from Konye and Kumba (Fig. 6a, *P* < 0.001) which was further reflected in mf intensities (Fig. 6b). Table 3 shows a detailed overview of each community. In our final analysis, we compared DERF communities that had had 0, 4 and > 10 years of IVM-MDA. Table 2 provides details about the health district Bare, which at the time of sampling had only participated in IVM-MDA for 4 years. Figure 6c, d shows that in comparison to communities that had received no IVM-MDA (*n* = 818 individuals, Table 1), both prevalence and intensity levels were lower in communities that had received 4 years of IVM MDA (*n* = 872 individuals, Table 2) and > 10 years of IVM-MDA (*n* = 3,672 individuals, Table 3). Thus, in confirmation of the longitudinal study shown in Fig. 4c, there is a general decrease in *M. perstans* prevalence and intensity in communities within DERF zones that have received 4–10 years of IVM-MDA (Additional file 3 Table S1).

## Discussion

The overall objective of this study was to provide a comprehensive overview about the current distribution of *M. perstans* in Cameroon, taking into consideration the advent of ivermectin MDA. Therefore, using a sample of 14,293 individuals from 137 communities, we determined the prevalence and intensity of *M. perstans* in five major bioecological zones of Cameroon. We observed that in the absence of MDA programmes, the highest prevalence of mansonellosis was restricted to equatorial rainforest areas since all three different savanna areas had only sporadic infection cases. Indeed, from 3,073 samples from savanna areas, only three individuals in Nkambe (GS), Kumbo (MFS) and Nwa (FS) were microfilaraemic. In contrast, *M perstans* was extremely prevalent in communities within DHERF and DERF zones with 20/29 communities showing prevalence over 50 %, 12/29 communities over 70 % and 3/29 communities over 90 %. Not a single community was found negative in the forest area. The lowest prevalence (12.8 %) was observed at Gabaleta in Batouri. The geometric mean of mf was also high reaching an average of 91,380.1 and 94,757.9 mf/ml in individuals in Ajaman and Mbofong, respectively, in Mamfe (DHERF). These data show that although *M. perstans* in Cameroon is widespread this species is largely restricted to ERF zones in which there can be hotspots of intensity. These results correlate with the observations of Akue et al. [18] in Gabon who also found that *M. perstans* was significantly higher in forest ecosystems than in savannas and lake land areas. Other studies have reported varying levels of *M. perstans* infection. For example, Noireau et al. reported a prevalence of 22 % in the forest regions of the Chaillu mountains in the Congo [19] and a prevalence of 26.6 % was observed in a community in the dense forest of southern Cameroon [13] but only 12.4 % of inhabitants in the forested Akonolinga health district of Central Cameroon were found to be *M. perstans*-positive [20]. Our findings of higher prevalence are more in-line with the studies from Asio et al. [21] who reported prevalence of up to 80 % in endemic communities of Uganda.

Cross-sectional surveys in communities of the study districts under treatment showed that although they had received varying rounds of IVM, a higher prevalence was still observed in DERF and DHERF compared to FS. The higher prevalence of *M. perstans* infection in forest areas (Mamfe, Messamena, Bertoua, Batouri, Lolodorf and some areas of Bare health districts) reflects the environmental conditions desired by the *Culicoides* spp. vectors. In contrast to GS and MFS areas, the DHERF and DERF zones provide ideal breeding conditions for the vectors. These include a warmer climate, sufficient rainfall and appropriate habitats such as fallen leaves, decaying plantains and banana stems. Moreover, these breeding sites are close to the hosts' dwellings providing an opportunity for parasite transmission. However, it can also not be ruled out that the species of *Culicoides* responsible for transmitting *M. perstans* is simply just refined to DHERF and DERF zones. Parallel entomology surveys were not performed with these surveys but future studies should concentrate on identifying the *Culicoides* spp. in those areas.

The intensity of infection as expressed by the geometric mean of microfilarial load varied across communities of the surveyed health districts. In the no treatment area, the mean ranged from 0 mf/ml in the GS of Nkambe health district through 13.9 mf/ml in the FS of Nwa to 17,382.2 mf/ml in the DHERF of Mamfe and closely followed by the DHERF of Lolodorf with 7,814.8 mf/ml. The same trend was observed with the prevalence in these areas. Moving across the five bioecological zones, the intensity increased from GS, through MFS (mosaic forest savanna) and FS to DERF and DHERF. Nevertheless, the high intensities in the DHERF before IVM MDA still emphasize the conserved nature of this ecological zone, making transmission very possible between the human host and the competent vector. A limitation of this study included the use of thick blood smears for *M. perstans* diagnosis which may have missed low mf counts. This diagnostic tool was employed so that all samplings from different time periods could be compared with another.

In the health districts under IVM MDA, the highest mean intensity (3,062.6 mf/ml) was observed in the DHERF of Bertoua with 8 years of ivermectin MDA and the lowest (175.8 mf/ml) in the FS of Nwa with equally 8 years of IVM MDA. The health districts under control for over 10 years had relatively lower intensities compared to those of 4 and 8 years. The decreasing trend in intensity observed over time in these bioecological zones with an increase in the number of rounds of IVM consumed is indicative that this drug has partial effect on *M. perstans* in the human host, although some authors have shown that the drug has no effect on the parasite [14, 20]. The varying intensity and prevalence in our study participants show that bio-ecology plays a defining role in the distribution of *M. perstans* even without the influence of ivermectin. To elucidate the effect of IVM-MDA on *M. perstans*, we performed a longitudinal survey using data from 7 communities in Mamfe (screened at 0 MDA and > 10 years IVM-MDA) as a case study. Our initial conclusion was that long-term IVM-MDA in the communities was having little effect on *M. perstans* infection since study sites showed both trends of increase and decrease. However, further analysis revealed that although the overall prevalence was not significantly reduced in the DERF communities (*P* = 0.335), the decrease in intensity over 10 years of MDA was significant (*P* < 0.001 and a 34.58 % reduction). This trend was not reflected in the DHERF communities of Mamfe since the prevalence either remained stagnant or had increased over time. Interestingly, mf levels in Nkoghau were drastically reduced after > 10 years MDA (11,391.1 *vs* 1,795.4 mf/ml) even though the prevalence were comparable (58.9 *vs* 63.3 %). Overall, our data indicate that in DERF communities of Mamfe, transmission is reduced due to either a loss of primary forest and thus breeding sites for the vector, due to impact of IVM, which needs at least 10 years to take effect, or possibly a lowered infection rate in the host. Since the reduction in *M. perstans* was not observed after long-term MDA in DHERF communities of Mamfe, the effects are more likely to be a consequence of habitat destruction. Studies relating *M. perstans* infection and IVM-MDA have shown contrasting outcomes. For example, Kyelem et al. [22] demonstrated that after multiple rounds of IVM-MDA in south-western Burkina Faso (Dano area), a significant reduction in *M. perstans* and *Wuchereria bancrofti* mf was seen in ivermectin-treated communities. However, a follow-up study by the same authors reported that long term administration (14 years) of IVM-MDA in Burkina Faso (Dienkoa) had no effects on *M. perstans* [23]. Of interest, this latter study was performed in a savanna ("Guinea" type) bioecological zone and there, prevalence of *M. perstans* infection (0–10.9 %) was higher than those reported in the present study (0–1.06 %) [23]. Research by other teams in Togo and Uganda [24, 25] have also not confirmed the trend of reduced infection rates in *M. perstans* following IVM seen by Kyelem et al. [22]. These studies were conducted in the early 90's and therefore only a few rounds of IVM treatment would have occurred since CDTI only commenced in 1995. Fischer et al. [24] reported that after IVM intake, prevalence rates were comparable but no data on mf intensity were provided. The study in Uganda [24] followed mf counts during a six month period after IVM administration and noted that prevalence rates remained the same but mf counts dropped by approximately 40 % within the first two weeks of treatment. Interestingly however, mf counts then stabilised and, since it remains unclear whether *M. perstans* has a microfilarial reservoir in the host, it cannot be stated whether mf are destroyed or eliminated [25]; this is an element that has to be factored into our findings as well. Furthermore, some of these studies were monitored in individuals co-infected with other filariae or in endemic areas for other filariae [22–25] and since IVM kills mf of both *Onchocerca volvulus* and *W. bancrofti*, immune responses to those dying mf may have some overlapping effects on *M. perstans* mf. In addition, studies have shown that *M. perstans* infections down-regulate the host's immune system through the involvement of IL-10 which may facilitate infections to other incoming pathogens [7, 8, 12]. Studies have investigated the impact of filarial infections on coincident intracellular pathogens and demonstrated the interplay between filarial parasite and microbial pathogen, but the influence of the interplay on disease outcome remains unclear [26]. Unlike other filarial infections, infection with *M. perstans* begins at childhood and the intensity increases with age [5, 27]. We therefore hypothesise that in areas of high *M. pertans* transmission, the immune response in children infected could be downregulated with negative consequences on the mounting of strong immune responses during childhood vaccinations and to control coincident pathogens. The present study has delineated the endemicity of *M. perstans* in the forest block of Cameroon and demonstrated that the ecology that favours the vector is of paramount importance. This gives an opportunity to design studies that can compare the immune responses of children to vaccination and other pathogens in contrasting areas of endemicity of *M. perstans*.

In the east and south, a concrete comparison and conclusion cannot be made for Batouri, Bertoua Messamena and Lolodorf health districts because no baseline data exist for the communities of the bioecological zones. Nevertheless, since the zones belong to the same ecological zone (DHERF), we see that at Batouri and Lolodorf that were not yet under CDTI (no treatment) the prevalence was 38.9 and 53.8 %, respectively, whereas those of Bertoua and Messamena (with 8 years of annual IVM) were 50.1 and 51 %, respectively. These high rates of infection in both non-treated and treated communities of the same bioecological zone may be due to (i) low adherence to treatment due to the presence of *L. loa* in these areas and consequent fear of severe adverse events; and (ii) little or no effect of IVM on the parasite although this will mean that the same communities be screened at 10 and 12 years after CDTI. Just as the prevalence increased in these zones, the intensity of infection also increased. This is normal if the drug is not effective, these parameters should increase with time. In the FS of Nwa health district, although the prevalence was low, a significant decrease was observed in the mf intensity after 8 years of IVM MDA. The consistent low prevalence in this zone could be due to the ecology of the area (lack of breeding sites as well as the vector) and the lack of heavy infection to propagate transmission, and not the fact that individuals here have received up to 8 rounds of mass treatment with ivermectin.

Comparison of DHERF zones in the east and south-west following multiple MDA rounds revealed a decrease in the prevalence in south-western but not in eastern Cameroon. The observed reduction in Mamfe may be due to: (i) better compliance in Mamfe than in Messamena and Bertoua; (ii) that at least 10 years of IVM treatment are required before MDA begins to have an effect on *M. perstans* infections; or (iii) changes or destruction of the forest zone in Mamfe over the last decade leading to reduced vector breeding sites and consequent abundance. The persistence of the infection in Messamena and Bertoua despite 8 years of IVM MDA could be due to poor adherence of the population to the MDA programmes as demonstrated by Wanji et al. [28].

## Conclusions

This study has demonstrated that *M. perstans* is highly endemic in the equatorial rainforest areas and low in savanna areas of Cameroon and this has not been affected by long-term IVM-MDA programmes. Before CDTI introduction, the high prevalence and intensities of *M. perstans* infection were observed in both DERF and DHERF of Mamfe. After a decade of IVM-MDA infection levels had dropped in individuals residing in DERF and not in DHERF communities. The decrease in infection intensity could be either due to IVM-MDA intervention or due to ecological changes over time that have led to a loss of breeding sites and a consequent decrease in the vectors’ abundance. Since it is known that *M. perstans* downregulates host's immune system, the findings from this work would be useful in designing studies to understand the impact of *M. perstans* on host immune response to vaccination and co-infection with other pathogens such as *Mycobacterium* spp. and *Plasmodium* spp. in areas of contrasting endemicities.

## Abbreviations

CDTI, Community directed treatment with ivermectin; DERF, deciduous equatorial rainforest; DHERF, dense humid equatorial rainforest; ERF, equatorial rainforest; FS, forested savannah; GS, grassland savanna; IVM, ivermectin; IVM-MDA, ivermectin mass drug administration; MDA, mass drug administration; Mf, microfilariae; MFS, mosaic forest savanna.

## Additional files

Additional file 1: Figure S1. Overview of gender distribution and median age in study sites. Out of 14,293 (6,746 males and 7,547 females) individuals involved in the study, 52.8 % were females. (PDF 224 kb) Additional file 2: Figure S2. Bioecological zones, health districts and test statistics performed (PDF 14 kb) Additional file 3: Table S1. Longitudinal study in Nwa confirms absence of *M. perstans* infection (a) Prevalence and (b) mf intensity in Nwa health district (10 communities) prior to IVM therapy (0 MDA; *n* = 1,029) and after 8 years of MDA programme (8 MDA; *n* = 1,329). In 2000, only 2 individuals positive and in 2013, 6 individuals were *M. perstans* positive. (DOCX 167 kb)
