# Supplementary figures and images for: Update on the distribution of Mansonella perstans in the southern part of Cameroon: influence of ecological factors and mass drug administration with ivermectin
Source: Parasit Vectors. 2016 May 31;9:311. doi: 10.1186/s13071-016-1595-1 (PMC4886396; doi:10.1186/s13071-016-1595-1)

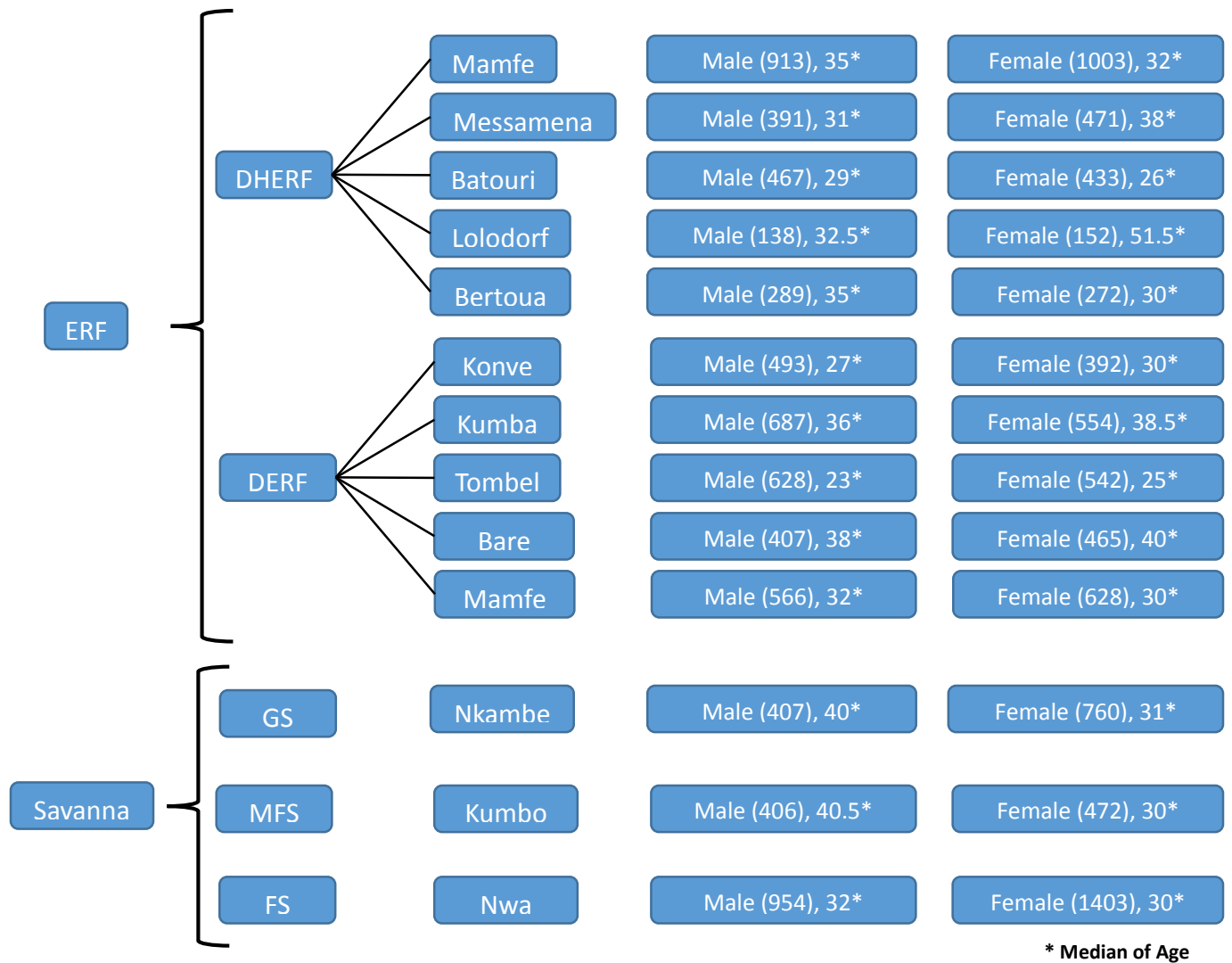

Additional file 1

Supplement: Additional file 1: Figure S1. — Overview of gender distribution and median age in study sites. Out of 14,293 (6,746 males and 7,547 females) individuals involved in the study, 52.8 % were females. (PDF 224 kb) [file 13071_2016_1595_MOESM1_ESM.pdf]

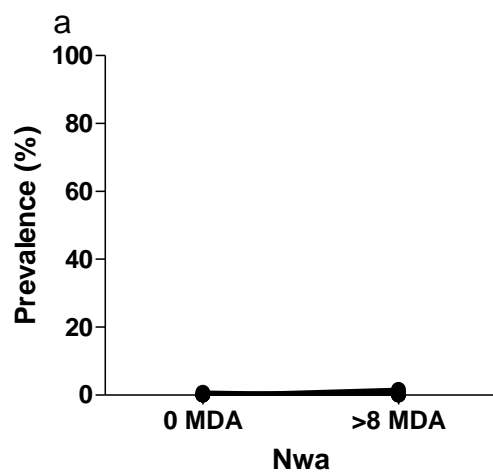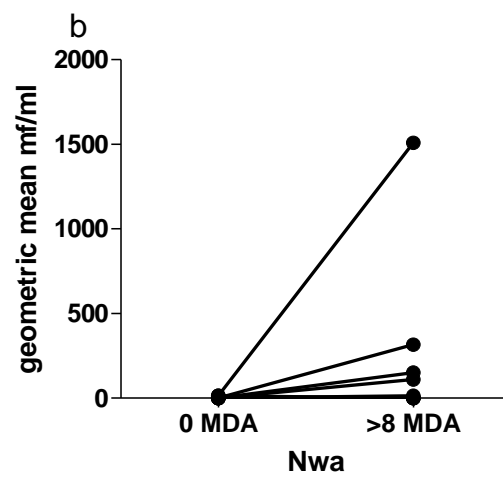

Additional file 2

Supplement: Additional file 2: Figure S2. — Bioecological zones, health districts and test statistics performed (PDF 14 kb) [file 13071_2016_1595_MOESM2_ESM.pdf]
