# Supplementary material for: Update on the distribution of Mansonella perstans in the southern part of Cameroon: influence of ecological factors and mass drug administration with ivermectin
Source: Parasit Vectors. 2016 May 31;9:311. doi: 10.1186/s13071-016-1595-1 (PMC4886396; doi:10.1186/s13071-016-1595-1)
Supplement: Additional file 3: Table S1. — Longitudinal study in Nwa confirms absence of M. perstans infection (a) Prevalence and (b) mf intensity in Nwa health district (10 communities) prior to IVM therapy (0 MDA; n = 1,029) and after 8 years of MDA programme (8 MDA; n = 1,329). In 2000, only 2 individuals positive and in 2013, 6 individuals were M. perstans positive. (DOCX 167 kb) [file 13071_2016_1595_MOESM3_ESM.docx]

**Additional file 3.**

| **Figure** | **Groups** | **Overall N** | **Overall prevalence** | **Applied statistical test** | ***P*-value** | **Overall geometric mean** | **Applied statistical test** | ***P*-value** |
| --- | --- | --- | --- | --- | --- | --- | --- | --- |
| **Comparison of Bioecological Zones and related Health Districts** | | | | | | | | |
| 3a/b | Savanna/ERF | 26/29 |  | MW t-test | <0.0001 |  | MW t-test | <0.0001 |
| 3c/d | FS/DERF | 10/22 | 0.2/69.4 | MW t-test | 0.0005 | 13.9/4,344.7 | MW t-test | 0.0006 |
| 3c/d | MFS/DERF | 8/7 | 0.11/69.4 | MW t-test | 0.0008 | 2,072.8/4,344.7 | MW t-test | 0.0019 |
| 3c/d | GS/DERF | 8/7 | 0/69.4 | MW t-test | 0.0010 | 0/4,344.7 | MW t-test | 0.0010 |
| 3c/d | FS/DHERF | 10/22 | 0.2/70.0 | MW t-test | <0.0001 | 13.90/17,382.9 | MW t-test | <0.0001 |
| 3c/d | MFS/DHERF | 8/22 | 0.11/70.0 | MW t-test | <0.0001 | 2,072.8/17,382.9 | MW t-test | <0.0001 |
| 3c/d | GS/DHERF | 8/22 | 0/70.0 | MW t-test | <0.0001 | 0/17,382.9 | MW t-test | 0.0001 |
| 3d/e | Mamfe/Lolodorf | 9/4 | 70.0/53.8 | MW t-test | 0.037 | 17,382.9/7,814.8 | MW t-test | 0.1986 |
| 3d/e | Mamfe/Batouri | 9/9 | 70.0/38.9 | MW t-test | 0.0020 | 17,382.9/1,947.7 | MW t-test | 0.0008 |
|  | | | | | | | | |
| **Comparison of DHERF Health Districts in relation to MDA** | | | | | | | | |
| 5a/b | 0 MDA/ >10 MDA | 13/13 |  | Unpaired t-test | 0.0091 |  | MW t-test | 0.0578 |
| 5a/b | >8 MDA/ >10 MDA | 16/13 |  | Unpaired t-test | 0.0002 |  | MW t-test | 0.0914 |
| 5b/d | Bertoua/Mamfe | 5/13 |  | Unpaired t-test | 0.0097 |  | MW t-test | 0.0611 |
| 5b/d | Messamena/Mamfe | 11/13 |  | Unpaired t-test | 0.0006 |  | MW t-test | 0.2710 |
| 5b/d | Lolodorf/Mamfe | 4/13 |  | Unpaired t-test | 0.0110 |  | MW t-test | 0.0475 |
| 5b/d | Mamfe/Mamfe_0_ | 13/9 |  | Unpaired t-test | <0.0001 |  | MW t-test | 0.0005 |
| 5b/d | Messamena/Mamfe_0_ | 11/9 |  | Unpaired t-test | 0.0106 |  | Unpaired t-test | 0.0125 |
| 5b/d | Batouri/Mamfe_0_ | 9/9 |  | Unpaired t-test | 0.0003 |  | Unpaired t-test | 0.0211 |
|  | | | | | | | | |
| **Comparison of DERF Health Districts in relation to MDA** | | | | | | | | |
| 6a/c | Mamfe/Tombel | 4/14 |  | MW t-test | 0.0068 |  | MW t-test | 0.1517 |
| 6a/c | Mamfe/Konye | 4/10 |  | MW t-test | 0.0020 |  | MW t-test | 0.0020 |
| 6a/c | Mamfe/Kumba | 4/17 |  | MW t-test | 0.0057 |  | MW t-test | 0.0143 |
| 6a/c | Tombel/Konye | 14/10 |  | MW t-test | 0.0192 |  | MW t-test | 0.0093 |
| 6a/c | Tombel/Kumba | 14/17 |  | MW t-test | 0.0010 |  | MW t-test | 0.0313 |
| 6c/d | 0 MDA/ 4 MDA | 7/12 |  | Unpaired t-test | 0.0160 |  | MW t-test | 0.0029 |
| 6c/d | 0 MDA/ >10 MDA | 7/45 |  | MW t-test | <0.0001 |  | MW t-test | 0.0003 |
|  | | | | | | | | |
